# Changes in Dietary Intake Patterns and Weight Status during the COVID-19 Lockdown: A Cross-Sectional Study Focusing on Young Adults in Malaysia

**DOI:** 10.3390/nu14020280

**Published:** 2022-01-10

**Authors:** Seok Tyug Tan, Chin Xuan Tan, Seok Shin Tan

**Affiliations:** 1 Department of Healthcare Professional, Faculty of Health and Life Sciences, Management and Science University, University Drive, off Persiaran Olahraga, Seksyen 13, Shah Alam 40100, Malaysia; sttan@msu.edu.my; 2 Department of Allied Health Sciences, Faculty of Science, Universiti Tunku Abdul Rahman, Jalan Universiti, Bandar Barat, Kampar 31900, Malaysia; tancx@utar.edu.my; 3 Department of Nutrition and Dietetics, School of Health Sciences, International Medical University, Bukit Jalil, Kuala Lumpur 57000, Malaysia

**Keywords:** dietary intake patterns, weight status, young adults, COVID-19

## Abstract

Introduction: The coronavirus disease 2019 (COVID-19) isolation has altered individuals’ food purchasing behaviour and dietary intake patterns. Therefore, this study aims to investigate the changes in dietary intake patterns and their impacts on the weight status of young adults in Malaysia during the COVID-19 lockdown. Methods: This cross-sectional study involved 1045 young adults in Malaysia. The changes in dietary intake patterns were assessed using the Dietary Diversity Questionnaire with slight modifications, while anthropometric measurements including body height, body weight before the pandemic and current body weight were self-reported. Results: Overall, nearly half of the respondents (48.8%) gained weight during the confinement, with an average increment of 4.06 ± 3.23 kg. Of 1045, 45.3% reported consuming more fruits and 60.2% had higher plain water intake during the pandemic. It is observed that 41.0% to 66.8% of the young adults changed their dietary intake patterns during the pandemic. Increased consumption in cereals and grains (β = 0.084, *p* = 0.015, 95% CI = 0.017–0.160), as well as oils and fats (β = 0.123, *p* = 0.001, 95% CI = 0.059–0.241), was positively associated with weight gain during the pandemic. On the contrary, an increased plain water intake was negatively associated with weight gain during the lockdown (β = −0.100, *p* = 0.003, 95% CI = −0.171–−0.034). Findings in the current study also suggested that cutting back cereals and grains (β = 0.156, *p* < 0.001, 95% CI = 0.122–0.288), as well as oils and fats (β = 0.091, *p* = 0.012, 95% CI = 0.022–0.183), contributed significantly to weight loss during the pandemic confinement. Conclusion: In conclusion, the enforcement of the Movement Control Order (MCO) drove up the prevalence of overweight/obesity among young adults in Malaysia. Increased consumption of cereals and grains and oils and fats contributed to weight gain in the pandemic lockdown. Nonetheless, a noticeable proportion of young adults in Malaysia shifted to a healthier food choice by increasing the consumption of fruits and vegetables.

## 1. Introduction

The coronavirus disease 2019 (COVID-19) has infected more than 200 million people globally as of August 2021 [1]. To date, close to 1.2 million Malaysians were infected by the COVID-19, in which the recent surge in confirmed cases was mainly driven by the highly transmissible Delta variant [2]. To curb the spread of COVID-19, the Federal Government of Malaysia decided to reinstate a nationwide lockdown or the Movement Control Order 3.0 (MCO 3.0) on 1 June 2021. The MCO 3.0 was the third nationwide lockdown, following the enforcement of MCO 1.0 from 18 March to 3 May 2020 and MCO 2.0 from 13 January to 4 March 2021 [3,4]. Several strict restrictions were implemented during the MCO 3.0; for instance, non-essentials sectors and learning institutions were ordered to temporarily shut down, only two in a family were allowed to do grocery shopping, and travel for necessities were limited to 10 km radius from an individual’s residential address [5].

A large body of literature demonstrated that COVID-19 isolation has altered individuals’ food purchasing behaviour and dietary intake patterns. People may deliberately reduce the frequency of grocery shopping, stock up non-perishable foods (including frozen, instant, processed or canned foods) and/or increase the frequency of home-cooking as strategies to diminish the risk of infection [6,7]. These changes eventually led to several mixed impacts on the dietary patterns, such as high level of sugars, salt and fats consumption from non-perishable foods, reduced intake of fresh foods (fruits, vegetables, seafood, fishes and livestock), or more reliance on healthier home-cooked meals [8,9,10]. In addition, emotional distress and boredom during the COVID-19 confinement also triggered a stronger desire for high-energy-dense foods that are rich in sugar and fat [11]. According to the recent National Health and Morbidity Survey 2019 (NHMS 2019), 50.1% of adults in Malaysia are either overweight or obese. Being female, Indian, and those in the range of 55–59 years old are particularly at a higher risk of being overweight or obese in Malaysia [12]. Apart from the aforementioned unhealthy dietary patterns, emotional eating and lack of physical activity during the pandemic isolation may also worsen the overweight and obesity crisis in Malaysia after the pandemic [11,13,14]. Therefore, this study aims to investigate the changes in dietary intake patterns and their impacts on the body weight status of young adults aged 18–30 years old in Malaysia throughout the COVID-19 home confinement.

## 2. Methodology

### 2.1. Study Design and Population

Data collection was conducted from 4–11 June 2021 (15 months after the first country lockdown was announced). A combination of convenience and snowball sampling approaches was adopted to recruit respondents into this study. A web-based anonymous self-administrated questionnaire was hosted on google forms and circulated to prospective respondents through social media platforms including WhatsApp, Facebook, Twitter, Instagram and TikTok. Informed consent was obtained from the respondents before revealing the first survey question. The sample size was estimated according to Krejcie and Morgan [15], assuming a 99% confidence level, 5% margin of error and 9 million young adults in Malaysia as of 2020 [16]. Therefore, the minimum sample size required for this study is 663 respondents. A total of 1055 respondents responded to this survey; however, findings in the current study were tabulated based on the responses from 1045 respondents living in Malaysia, after excluding those who do not fit the age of young adults (more than 30 years old), duplicate responses, and non-Malaysian. Ethical approval was obtained from the Research Ethics Committee of Management and Science University with the reference number MSU-RMC-02/FR01/06/L1/041.

### 2.2. Socio-Demographic Characteristics and Self-Reported Weight Change

Socio-demographic information, including gender, age, ethnicity, marital status, and educational attainment was self-reported by the respondents. The pre-pandemic body weight (kg) was retrospectively recalled by the respondents, while the current body weight (kg) was obtained by instructing the respondents to perform self-measurement using a bathroom scale whenever possible. Weight change was calculated by the difference in the current and pre-pandemic body weights [17]. In addition to the body weight, all respondents were required to report their body height (cm) for the estimation of body mass index (BMI) (kg/m^2^). The BMI was further stratified into underweight (<18.5 kg/m^2^), normal (18.5–22.9 kg/m^2^), overweight (23.0–24.9 kg/m^2^) and obese (≥25.0 kg/m^2^) according to the Asia-Pacific cut-off points [18].

### 2.3. Changes in Dietary Intake Patterns (∆Dietary Intake Patterns) throughout the Lockdown

Changes in dietary intake patterns were assessed using the dietary diversity questionnaire with slight modifications [19]. The original questionnaire consists of 17 food groups intended to examine the food consumption patterns at household and individual levels. This 17-item was matched with the newly developed Malaysian Food Pyramid 2020 to identify food groups that can reflect the local food consumption patterns [20]. Consequently, 13 food groups were included in the current study. Two additional items (salt and plain water intakes) were added to the questionnaire to represent all food groups/items listed in the Malaysian Food Pyramid 2020. The finalised questionnaire contains 15 items with three scales (reduced consumption, remained the same, increased consumption) (Table S1). All respondents were instructed to choose one scale which best represented their current dietary intakes compared to pre-pandemic.

### 2.4. Statistical Analysis

Data analysis was carried out using IBM SPSS version 26.0 (IBM Corp, Armonk, Westchester, NY, USA). Exploratory factor analysis (EFA) and Cronbach’s alpha were used to test the internal consistency and reliability of all items of the modified the dietary diversity questionnaire. When applicable, the frequency, percentage, mean and standard deviation (SD) were used to describe socio-demographic characteristics, weight status, BMI and changes in dietary intake patterns, throughout the COVID-19 pandemic. Continuous variables including age, body weight (kg) and BMI (kg/m^2^) before and during the pandemic were subjected to a normality test. These variables were considered normally distributed if the skewness was ±2. Mean difference in BMI before and during the pandemic was assessed with a paired samples *t*-test. Partial correlations with the adjustment of gender, age, ethnicity, marital status and educational attainment were applied to determine the relationship between dietary intake patterns (∆dietary intake patterns) and body weight (∆body weight) of the respondents throughout the pandemic home confinement. A food group that portrays a *p*-value of less than 0.25 (*p* < 0.25) in partial correlation was subsequently included in a hierarchical multiple regression model for analysis. A *p*-value of less than 0.05 (*p* < 0.05) was statistically significant.

## 3. Results

Exploratory factor analysis (EFA) using principal component analysis (PCA) was used to investigate construct validity of the modified dietary diversity questionnaire. The Kaiser–Meyer–Olkin index (KMO) was 0.823, with a statistically significant Bartlett’s sphericity test (χ^2^ = 1895.63, *p* < 0.001), indicating that the assumptions of conducting factor analysis was met. Based on the results of PCA, all items were retained in the final model and the cumulative variance accounted for 59.19%. The reliability of the modified dietary diversity questionnaire was good, with a Cronbach’s alpha of 0.810.

Table 1 shows the socio-demographic characteristics and weight status of the young adults during the COVID-19 lockdown. The majority were female (63.8%), aged 18–24 (80.7%), Malay (59.4%), with single marital status (94.4%) and tertiary educated (85.2%). Nearly half of the respondents (48.8%) gained weight during the confinement, with an average increment of 4.06 ± 3.23 kg. Interestingly, 36.3% of the young adults lost weight in the course of pandemic lockdown, with an average weight loss of 4.90 ± 4.38 kg. While there was no significant difference in the BMI before (22.78 ± 4.87 kg/m^2^) and during the pandemic (22.85 ± 4.70 kg/m^2^) (*t* = −1.215, *p* = 0.225), an increment in the BMI by 0.31% throughout the confinement was noted.

Table 2 demonstrates the changes in dietary intake patterns of young adults after 15 months of the COVID-19 outbreak in Malaysia. Of 1045, 45.3% reported consuming more fruits and 60.2% had higher plain water intake during the pandemic. Although the consumption patterns for the rest of the food groups remained relatively the same throughout the pandemic, there was also a noticeable proportion of young adults with increased intakes of eggs (43.3%), dark green vegetables (36.6%), milk and dairy products (36.4%), other vegetables (35.9%), cereals and grains (35.3%), flesh meats (35.1%), sugars and sweets (34.8%), vitamin A-rich tubers (34.4%), salts (31.9%), fish and shellfish (28.9%), as well as oils and fats (21.6%). Depending on the food groups/item, 41.0% (white tubers and roots) to 66.8% (plain water) of the young adults changed their dietary intake patterns (either consuming more or less in quantity) during the pandemic as opposed to pre-pandemic.

Table 3 depicts the partial correlations between ∆ dietary intake patterns and weight gain during the COVID-19 lockdown. Emerging results indicated that weight gain was positively correlated with increased cereals and grains (r_partial_ = 0.105, *p* = 0.001), oils and fats (r_partial_ = 0.140, *p* < 0.001), as well as sugars and sweets (r_partial_ = 0.085, *p* = 0.006) intakes during the confinement. As previously mentioned, food groups/items which portrayed *p* < 0.25 in partial correlation analysis were selected as the predictors for weight gain in hierarchical multiple regression. Therefore, cereals and grains, flesh meats, eggs, oils and fats, sugars and sweets, salts and plain water were included in the regression model. Table 4 shows the selected predictors (food groups/items) for weight gain during the COVID-19 lockdown. Increased consumption in cereals and grains (β = 0.084, *p* = 0.015, 95% CI = 0.017–0.160), as well as oils and fats (β = 0.123, *p* = 0.001, 95% CI = 0.059–0.241), was positively associated with weight gain during the pandemic. Another interesting finding worth mentioning is that an increased plain water intake was negatively associated with weight gain during the lockdown (β = −0.100, *p* = 0.003, 95% CI = −0.171–−0.034) (Table 4).

Table 5 shows the partial correlations between ∆ dietary intake patterns and weight loss during the COVID-19 pandemic. Cereals and grains (r_partial_ = 0.184, *p* < 0.001), legumes, nuts and seeds (r_partial_ = 0.049, *p* = 0.115), flesh meats (r_partial_ = 0.069, *p* = 0.027), milk and dairy products (r_partial_ = 0.051, *p* = 0.097), oils and fats (r_partial_ = 0.144, *p* < 0.001), as well as sugars and sweets (r_partial_ = 0.110, *p* < 0.001), were selected as the predictors for weight loss. Findings from hierarchical multiple regression indicated that reduced consumption in cereals and grains (β = 0.156, *p* < 0.001, 95% CI = 0.122–0.288), as well as oils and fats (β = 0.091, *p* = 0.012, 95% CI = 0.022–0.183), contributed significantly to weight loss during the pandemic confinement (Table 6).

## 4. Discussion

The Malaysian Food Pyramid was revised in 2020 to provide a better visual healthy eating guide to the public. It comprises of four levels corresponding to five food groups: fruits and vegetables (base/level 1), cereals, grains and tubers (level 2), poultry/eggs/meats, fish and legumes (level 3), milk and dairy products (level 3), as well as fats, oils, sugars and salt (level 4). The serving size of each food group is getting smaller when shifting from the base to the apex of the food pyramid, signalling that an individual should eat plenty of fruits and vegetables (level 1), while limiting the intakes of fats, oils, sugars and salt (level 4). Moreover, another important feature of the revised Malaysian Food Pyramid 2020 is that the recommendation for plain water intake is emphasized in the food pyramid [20]. To the best of the authors’ knowledge, the current study was the first to evaluate the dietary intake patterns with reference to the Malaysian Food Pyramid 2020.

Findings in this study revealed that the prevalence of overweight/obesity increased from 39.8% (pre-pandemic) to 41.5% (during the pandemic), with an increment of 1.7% due to the COVID-19 confinement. Moreover, 48.8% of the young adults had an average weight gain of 4.06 ± 3.23 kg after 15 months of the COVID-19 outbreak in Malaysia. A previous study by Tan, Tan, and Tan [13] indicated that Malaysian university students gained 3.9 kg in the first four months of pandemic confinement. By comparison, the magnitude of weight gain in the current study was slightly higher than those previously reported. Unhealthy dietary patterns, physical inactivity, and increased screen-time-based sedentary behaviour could be among the few contributing factors for weight gain during the COVID-19 lockdown [13,17,21].

Dietary intake patterns are generally shaped by income, individual preferences and beliefs, food prices and food environments [22]. Emerging findings showed that the consumption patterns for nearly all food groups remained relatively the same throughout the pandemic, suggesting that the food supply chains in Malaysia were remarkably resilient in COVID-19. One of the initiatives taken by the Malaysian Federal government to ensure sufficient food supply in the local market was to allow the agricultural, husbandry and fisheries sectors to continue operating during the third country lockdown (MCO 3.0) [23]. This appears as the underlying reason for the resilient food supply chains in the COVID-19 pandemic.

Literature has consistently reported that fruits and vegetables contain abundant amounts of phytochemicals and vitamins, which are essential for preventing non-communicable diseases [24,25]. Having sufficient fruit and vegetable intakes is also crucial for a well-functioning immune system in this unprecedented pandemic [26]. Prior to COVID-19, almost all Malaysian adults (95%) do not meet the recommendation of consuming at least five servings of fruits and vegetables in a day [12]. However, over the pandemic confinement, close to half (45.3%) increased fruit intake and one-third (35.9–36.6%) consumed vegetables more frequently. These findings suggest that the COVID-19 led to a greater willingness to consume fruits and vegetables.

The enforcement of COVID-19 home confinement did not significantly change the consumption patterns of carbohydrate and protein-rich foods (level 2 and level 3). The proportion of young adults declaring consumption of more carbohydrate-rich foods varied from 17.3% (white tubers and roots) to 35.3% (cereals and grains); meanwhile, those with increased consumption of cereals and grains (rice, noodles, pasta and bread) were more likely to gain weight during the pandemic. These findings are generally in line with a recent study by Pellegrini et al. [27], which reported that the increased consumption of cereals in the first month of pandemic lockdown was associated with weight gain among Italians. On the other hand, 20.8% (legumes) to 43.3% (eggs) of the young adults claimed to consume more protein-rich foods than pre-pandemic. When comparing these findings to those reported by Grant, Scalvedi, Scognamiglio, Turrini, and Rossi [28], it must be pointed out that the Italian adults attained a smaller magnitude of increment in red meat (10.7%), white meat (12.6%), as well as fish and shellfish (14.0%) intakes during the pandemic than the young adults in Malaysia. On the contrary, the proportion of those with increased consumption of legumes was comparable between young adults in Malaysia (20.8%) and Italian adults (22.1%).

There are numerous studies suggesting that boredom arising from the COVID-19 confinement spiked the consumption of comfort foods which are generally high in fats, sugars and salt [29,30,31]. The current study demonstrated that 21.6% to 34.8% of young adults in Malaysia consumed a larger portion of oily, sugary and savoury foods over the pandemic. Of these condiments, only oils and fats were associated with weight gain. It is also worth mentioning that young adults in Malaysia showed a satisfactory habit in plain water intake throughout the pandemic, with 60.2% drinking more water compared to pre-pandemic. This is consistent with what has been previously reported in the National Health and Morbidity Survey 2019 (NHMS 2019), indicating that 3 in 4 Malaysian adults drink sufficient plain water daily [12]. In addition, findings in the current study revealed that plain water consumption was inversely associated with weight gain during the lockdown. This finding is directly in-line with a recent study by García et al. [32], who reported that staying hydrated helps to maintain a healthy body weight. Besides, the current study also determined the contributing factors to weight loss during the COVID-19 pandemic. Emerging findings suggested that cutting back on cereals, grains, oils and fats were among the effective strategies to lose weight in the time of pandemic lockdown.

Findings presented in the current study must be interpreted within the context of limitations. Given that the current study adopted a cross-sectional study design, the reported findings might not be able to postulate the direct cause–effect relationships of dietary intake patterns and body weight status throughout the pandemic lockdown. The current study also acknowledged that self-reported anthropometric measurements may potentially be subject to reporting bias. Even though it is widely accepted that the Food Frequency Questionnaire (FFQ) and multiple 24-h dietary recalls provide a better estimation on dietary intake patterns, these assessment tools require a well-trained nutritionist/dietitian in acquiring necessary information from the subjects [33]. In view of the enactment of physical distancing order, the current study decided to adopt a simple and quick approach to get to know the dietary patterns of young adults in Malaysia over the pandemic confinement. Besides, it is also noted that web-based surveys had apparently ruled out those without internet access in the pandemic or economically disadvantaged groups. Therefore, the current study’s findings might not sufficiently represent the dietary intakes of all young adults in Malaysia during the pandemic. Nevertheless, this was the first study that investigated the changes in dietary intake patterns and body weight status of young adults in Malaysia throughout the COVID-19 pandemic.

## 5. Conclusions

The enforcement of MCO drove up the prevalence of overweight/obesity among young adults in Malaysia. The consumption of cereals and grains, as well as oils and fats, determined weight change in the pandemic lockdown. As obesity is linked to a greater likelihood of developing non-communicable diseases including hypertension, type II diabetes and cancers in later life [34], findings in the current study can provide insights to develop relevant weight management strategies for young adults after the pandemic. Moreover, future study should also consider the potential interactions between physical activity and dietary intake patterns towards body weight status. Despite those previously mentioned, a noticeable proportion of young adults in Malaysia shifted to a healthier food choice through increasing the consumption of fruits and vegetables for a robust immune system during the COVID-19 pandemic.

## Figures and Tables

**Table 1:** Socio-demographic characteristics and weight status of the respondents during the COVID-19 lockdown.

Variable	Frequency, *n* (%)	Mean ± Standard Deviation
Gender		-
Male	378 (36.2)	
Female	667 (63.8)	
Age (years old)		22.66 ± 2.47
18–24	843 (80.7)	
25–30	202 (19.3)	
Ethnicity		-
Malay	621 (59.4)	
Chinese	170 (16.3)	
Indian	230 (22.0)	
Others	24 (2.3)	
Marital status		-
Single	978 (94.4)	
Married	50 (4.8)	
Divorced/Widowed	8 (0.8)	
Educational attainment		-
No formal education/Primary	18 (1.7)	
Secondary	137 (13.1)	
Tertiary	890 (85.2)	
Weight status (kg)		
Sustained weight	156 (14.9)	0
Weight loss	379 (36.3)	−4.90 ± 4.38
Weight gain	510 (48.8)	4.06 ± 3.23
BMI (kg/m^2^)		
Before the pandemic		
Underweight	186 (17.8)	
Normal	443 (42.4)	22.78 ± 4.87 ^a^
Overweight	159 (15.2)	
Obese	257 (24.6)	
During the pandemic		
Underweight	162 (15.5)	
Normal	450 (43.1)	22.85 ± 4.70 ^a^
Overweight	166 (15.9)	
Obese	267 (25.6)	

^a^ Mean difference was tested with a paired samples *t*-test. Different letters indicate significant difference at *p* < 0.05.

**Table 2:** Changes in dietary intake patterns of young adults in Malaysia during the COVID-19 lockdown.

Food Groups/Items	∆Dietary Intakes		
	Reduced Intake, *n* (%)	Remained the Same, *n* (%)	Increased Intake, *n* (%)
Level 1			
Fruits	159 (15.2)	413 (39.5)	473 (45.3)
Vegetables			
Dark green vegetables	153 (14.6)	510 (48.8)	382 (36.6)
Other vegetables	134 (12.8)	536 (51.3)	375 (35.9)
Level 2			
Cereals and grains	165 (15.8)	511 (48.9)	369 (35.3)
Tubers			
White tubers and roots	247 (23.7)	617 (59.0)	181 (17.3)
Vitamin A-rich tubers	162 (15.5)	523 (50.1)	360 (34.4)
Level 3			
Legumes	238 (22.8)	590 (56.4)	217 (20.8)
Fish and shellfish	206 (19.7)	537 (51.4)	302 (28.9)
Flesh meats	157 (15.0)	521 (49.9)	367 (35.1)
Eggs	106 (10.1)	487 (46.6)	452 (43.3)
Milk and dairy products	191 (18.3)	474 (45.3)	380 (36.4)
Level 4			
Oils and fats	247 (23.6)	572 (54.8)	226 (21.6)
Sugars and sweets	277 (26.5)	404 (38.7)	364 (34.8)
Salts	177 (16.9)	535 (51.2)	333 (31.9)
Plain water	69 (6.6)	347 (33.2)	629 (60.2)

**Table 3:** Partial correlation between ∆ dietary intake patterns and weight gain during the COVID-19 lockdown.

Food Groups/Items ^1^	Partial Correlation, r_partial_ ^2^	*p*-Value ^3^
Level 1		
Fruits	0.007	0.818
Vegetables		
Dark green vegetables	−0.027	0.393
Other vegetables	−0.017	0.589
Level 2		
Cereals and grains	0.105	0.001
Tubers		
White tubers and roots	0.028	0.370
Vitamin A-rich tubers	−0.027	0.376
Level 3		
Legumes, nuts and seeds	0.002	0.943
Fish and shellfish	0.029	0.356
Flesh meats	0.052	0.095
Eggs	0.050	0.107
Milk and dairy products	0.031	0.313
Level 4		
Oils and fats	0.140	<0.001
Sugars and sweets	0.085	0.006
Salts	0.039	0.206
Plain water	−0.053	0.088

^1^ Dietary intakes were dummy coded as 0 = reduced intakes/remained the same and 1 = increased intakes. ^2^ Partial correlation with the adjustment of gender, age, ethnicity, marital status and educational attainment. ^3^ Variable which portrays a *p*-value of less than 0.25 (*p* < 0.25) was included into hierarchical multiple regression model.

**Table 4:** Predictors of weight gain during the COVID-19 lockdown.

∆Dietary Intakes ^1^	B (SE)	β	*p*-Value ^2^	95% CI
Level 2				
Cereals and grains	0.088 (0.036)	0.084	0.015	0.017–0.160
Level 3				
Flesh meats	0.009 (0.040)	0.009	0.815	−0.069–0.087
Eggs	0.024 (0.038)	0.024	0.533	−0.051–0.099
Level 4				
Oils and fats	0.150 (0.046)	0.123	0.001	0.059–0.241
Sugars and sweets	0.020 (0.038)	0.019	0.596	−0.055–0.096
Salts	−0.042 (0.040)	−0.039	0.293	−0.121–0.036
Plain water	−0.102 (0.035)	−0.100	0.003	−0.171–−0.034

^1^ With the adjustment of gender, age, ethnicity, marital status and educational attainment. ^2^ Significant difference was considered at *p* < 0.05.

**Table 5:** Partial correlation between ∆ dietary intake patterns and weight loss during the COVID-19 lockdown.

Food Groups/Items ^1^	Partial Correlation, r_partial_ ^2^	*p*-Value ^3^
Level 1		
Fruits	0.014	0.661
Vegetables		
Dark green vegetables	−0.010	0.746
Other vegetables	−0.005	0.859
Level 2		
Cereals and grains	0.184	<0.001
Tubers		
White tubers and roots	0.016	0.598
Vitamin A-rich tubers	−0.004	0.889
Level 3		
Legumes, nuts and seeds	0.049	0.115
Fish and shellfish	0.026	0.405
Flesh meats	0.069	0.027
Eggs	0.025	0.420
Milk and dairy products	0.051	0.097
Level 4		
Oils and fats	0.144	<0.001
Sugars and sweets	0.110	<0.001
Salts	0.025	0.419
Plain water	−0.014	0.650

^1^ Dietary intakes were dummy coded as 0 = increased intakes/remained the same and 1 = reduced intakes. ^2^ Partial correlation with the adjustment of gender, age, ethnicity, marital status and educational attainment. ^3^ Variable which portrays a *p*-value of less than 0.25 (*p* < 0.25) was included into hierarchical multiple regression model.

**Table 6:** Predictors of weight loss during the COVID-19 lockdown.

∆Dietary Intakes ^1^	B (SE)	β	*p*-Value ^2^	95% CI
Level 2				
Cereals and grains	0.205 (0.042)	0.156	<0.001	0.122–0.288
Level 3				
Legumes, nuts and seeds	0.023 (0.038)	0.020	0.542	−0.051–0.098
Flesh meats	−0.006 (0.045)	−0.004	0.902	−0.095–0.083
Milk and dairy products	−0.020 (0.042)	−0.016	0.645	−0.103–0.064
Level 4				
Oils and fats	0.103 (0.041)	0.091	0.012	0.022–0.183
Sugars and sweets	0.039 (0.038)	0.036	0.296	−0.035–0.113

^1^ With the adjustment of gender, age, ethnicity, marital status and educational attainment. ^2^ Significant difference was considered at *p* < 0.05.

## Supplementary Materials

The following supporting information can be downloaded at: https://www.mdpi.com/article/10.3390/nu14020280/s1, Table S1: The Modified Dietary Diversity Questionnaire.
