# Supplementary material for: Changes in Dietary Intake Patterns and Weight Status during the COVID-19 Lockdown: A Cross-Sectional Study Focusing on Young Adults in Malaysia
Source: Nutrients. 2022 Jan 10;14(2):280. doi: 10.3390/nu14020280 (PMC8778075; doi:10.3390/nu14020280)
Supplement: Supplementary file 1 [file nutrients-14-00280-s001.zip › nutrients-1517388-supplementary.pdf]

Table S1. The Modified Dietary Diversity Questionnaire.

Instruction: This section aims to assess the food consumption patterns before and during the COVID-19 lockdown. Please select **ONE** option which best represented your food intakes.

Question: During the COVID-19 lockdown, my consumption for **(substitute with item from column A)** *reduced/remained the same/increased* compared to pre-pandemic.

For example: During the COVID-19 lockdown, my consumption for **cereals and grains** *reduced/remained the same/increased* compared to pre-pandemic.

Item (column A)	Examples	Food consumption pattern [1]		
		Reduced	Remained the same	Increased
Cereals and grains	Rice, corn, wheat. bread, noodles, pasta, porridge, oats, grains, etc.			
Vitamin A-rich tubers	Carrots, sweet potatoes, etc.			
White tubers and roots	White potatoes, white yam, white cassava, etc.			
Dark green leafy vegetables	*Bayam* (spinach), *sawi* (Mustard greens), *kangkong* (Water Spinach), etc.			
Other vegetables	Tomato, onion, eggplant, etc.			
Fruits	Apple, oranges, mangoes, apricots, papaya, peaches, guava, durian, *rambutan*, etc.			
Flesh meats	Beef, pork, lamb, chicken, duck, etc.			
Eggs	Chicken egg, duck egg, preserved egg, etc.			
Fish and shellfish	Fresh/dried/canned fish, shellfish, etc.			
Legumes, nuts and seeds	Beans, peas, lentils, nuts, seeds, or foods made from these.			
Milk and dairy products	Milk, cheese, yogurt, etc.			
Oils and fats	Oils, fats or butter added to food or used for cooking.			
Sugars and sweets	Sugar, honey, carbonated drinks, chocolates, candies, cookies and cakes.			
Salts	Processed foods, salty snacks or salts added to food or used for cooking.			

Plain water	Boiled water, bottled water (drinking water/mineral water/unsweetened sparkling water), etc.			

[1] Respondents were only allowed to select one option in Google forms.
